# Virulence factors of *Streptococcus anginosus* – a molecular perspective

**DOI:** 10.3389/fmicb.2022.1025136

**Published:** 2022-10-26

**Authors:** Aleksandra Kuryłek, Monika Stasiak, Izabela Kern-Zdanowicz

**Affiliations:** Laboratory of Bacterial Drug Resistance, Institute of Biochemistry and Biophysics Polish Academy of Sciences, Warsaw, Poland

**Keywords:** SAG, *Streptococcus milleri*, commensal, opportunistic infections, invasive infections

## Abstract

*Streptococcus anginosus* together with *S. constellatus* and *S. intermedius* constitute the *Streptococcus anginosus* group (SAG), until recently considered to be benign commensals of the human mucosa isolated predominantly from oral cavity, but also from upper respiratory, intestinal, and urogenital tracts. For years the virulence potential of SAG was underestimated, mainly due to complications in correct species identification and their assignment to the physiological microbiota. Still, SAG representatives have been associated with purulent infections at oral and non-oral sites resulting in abscesses formation and empyema. Also, life threatening blood infections caused by SAG have been reported. However, the understanding of SAG as potential pathogen is only fragmentary, albeit certain aspects of SAG infection seem sufficiently well described to deserve a systematic overview. In this review we summarize the current state of knowledge of the *S. anginosus* pathogenicity factors and their mechanisms of action.

## Introduction

**Figure 1:**
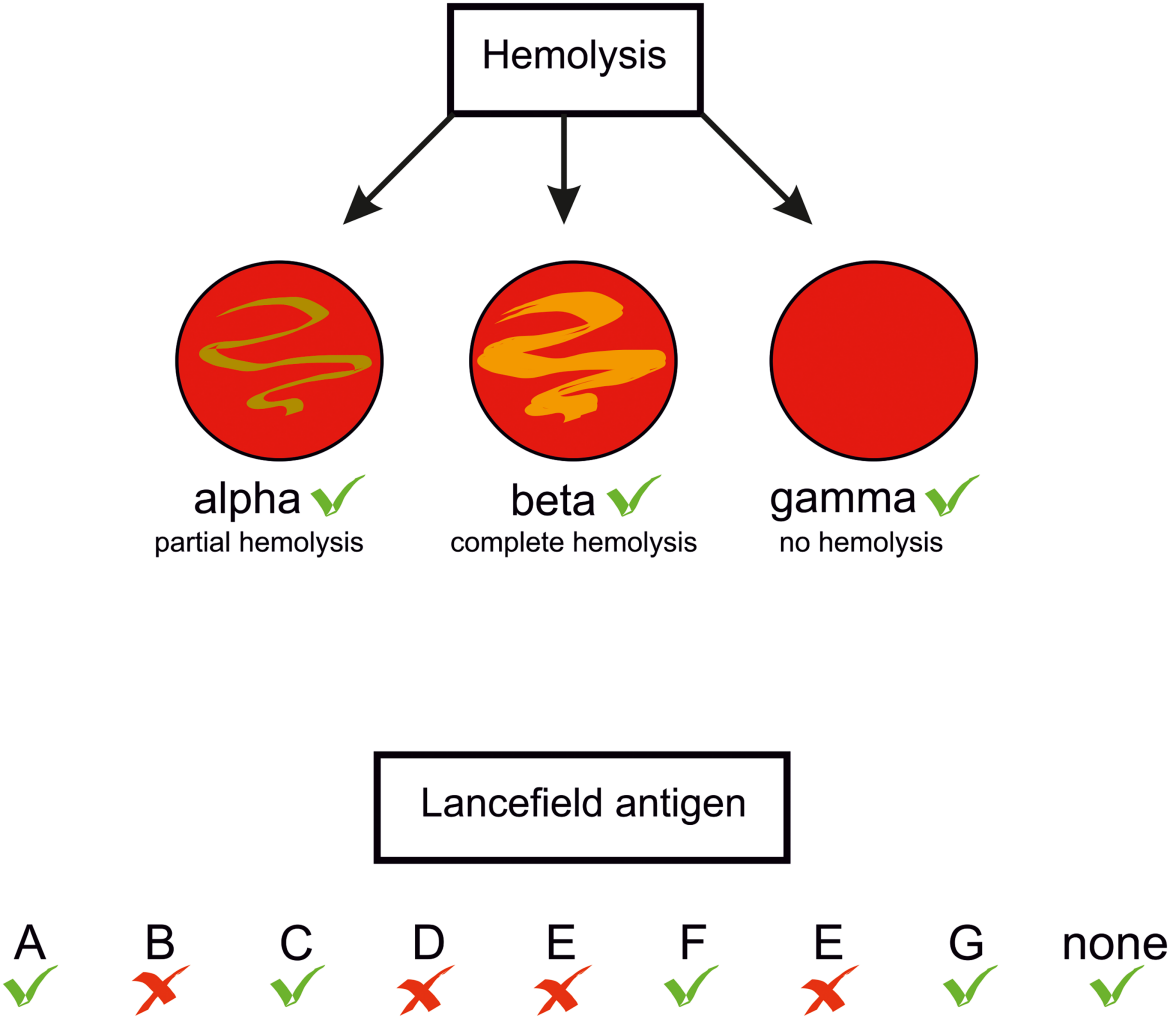
Types of hemolysis and Lancefield antigens presented by *S. anginosus* strains. Green tick –feature present in some *S. anginosus* strains, red cross – absent.

In the genus *Streptococcus* the species *S. pyogenes*, *S. agalactiae*, *S. pneumoniae*, and *S. dysgalactiae* subsp. *equisimilis* (SDSE) are most frequently responsible for morbidity and mortality in humans (Carapetis et al., 2005; O’Brien et al., 2009; Rantala, 2014; Lawn et al., 2017). However, other streptococci, previously considered just human commensals, are recognised as a cause of human diseases, e.g., oral streptococci *S. sanguinis* (Vogkou et al., 2016; Zhu et al., 2018), *S. intermedius* (Sadykov et al., 2003; Mishra and Fournier, 2013; Issa et al., 2020), *S. constellatus* (Akuzawa et al., 2017), and *S. anginosus* (Clarridge et al., 2001; Asam and Spellerberg, 2014). Clinical data indicate that also *S. suis* and *S. iniae*, porcine and fish pathogens, respectively, can infect humans (Weinstein et al., 1997; Lun et al., 2007; Baiano and Barnes, 2009). Distinguishing between pathogenic and non-pathogenic strains was historically based on their ability to lyse erythrocytes on blood-agar plates: those forming a clear zone of hemolysis (β-hemolysis), were considered potentially pathogenic. A further differentiation was proposed in 1933 by Rebecca Lancefield, who identified different versions of the major cell wall polysaccharide of streptococci, later called the group polysaccharide or the Lancefield antigen. According to this classification, the human pathogens *S. pyogenes* and *S. agalactiae* belong to group A (GAS) and group B (GBS) streptococci, respectively. Other streptococci are not homogenous in expressing the Lancefield antigens, e.g., *S. anginosus* strains can bear group F, C, G, or even group A antigens, or none (Obszańska et al., 2016; Baracco, 2019; Figure 1). Rather than basing on the antigens, the currently accepted classification of streptococci reflects their phylogenetic relationships. Accordingly, they are classed into seven groups: pyogenic, anginosus, mitis, salivarius, bovis, mutans, and species of unknown position, as for example *S. suis* (Köhler, 2007). *S. anginosus*, *S*. *constellatus* and *S. intermedius* constitute the *Streptococus anginosus* group (SAG, the Anginosus group). Formerly, these three species were regarded by European and Japanese microbiologists as a single species “*Streptococcus milleri*.” However, they were distinguished by microbiologists from North America. [reviewed in (Coykendall, 1989) and recently in (Pilarczyk-Zurek et al., 2022)]. Previously, SAG was classed in a diverse group of streptococci called the viridans group which was poorly characterized(Coykendall, 1989). The name “viridans” comes from the Latin “viridis” meaning “green” and was given to the bacteria due to a greenish halo around their colonies on blood agar. The green or brown colouring results from the oxidation of hemoglobin to methemoglobin and is called α-hemolysis. However, the name viridans was misleading, as not all *S*. *anginosus* strains present this type of hemolysis: some are β-hemolytic and some are even nonhemolytic, in other words – they present γ-hemolysis, a confusing term referring to a lack of hemolysis (Coykendall, 1989; Whiley et al., 1990; Fox et al., 1993; Broyles et al., 2009; Fischetti and Ryan, 2015; Whiley and Hardie, 2015; Figure 1). In 2013, a further differentiation of SAG was proposed on the basis of the nucleotide sequences of seven housekeeping genes: *S. constellatus* was divided into three subspecies, *constellatus*, *pharyngis*, and *viborgensis*, and *S. anginosus* into two subspecies, *anginosus* and *whileyi*; *S. intermedius* remained as a single species (Jensen et al., 2013). The type strain *S. anginosus* NCTC 10713 ( = ATCC 33397, ATCC 12395), a β-hemolytic strain with the Lancefield group G antigen isolated from human throat, is classified as *S. anginosus* subsp. *anginosus*. It should be noted here that detection of Lancefield antigen and biochemical tests used in laboratory practice for SAG identification, and even mass spectrometry (MALDI-TOF-MS), do not allow a reliable identification at species level and the results should be viewed with caution (Whiley et al., 1990; Wenzler et al., 2015). The precise SAG species identification can be achieved with the use of the molecular biology-based methods; the SAG identification issues have been recently reviewed in (Pilarczyk-Zurek et al., 2022).

In the past all SAG species were considered to be human commensals, part of the healthy human microbiota, commonly residing on mucosal membranes of the oral cavity, preferentially found in dental plaques, but also on mucosal membranes of the gastrointestinal, upper respiratory, and urogenital tracts (Hamada and Slade, 1980; Whiley et al., 1992). However, more and more data link SAG members with health problems in humans, especially in immunocompromised, cancer, or cystic fibrosis patients [reviewed in (Pilarczyk-Zurek et al., 2022)]. According to observations, the clinical syndromes differed depending on the SAG species involved: *S. constellatus* was associated with odontogenic, soft tissue, pleuropulmonary, and intra-abdominal abscesses, *S. intermedius* with pleuropulmonary infections, abscesses of brain, and deep soft tissues, whereas *S. anginosus* was rarely responsible for abscesses and more frequently than two other SAGs was isolated from blood, infected soft tissues, and urine (Whiley et al., 1992; Clarridge et al., 2001; Junckerstorff et al., 2014; Issa et al., 2020; Jiang et al., 2020). Nevertheless, there are several reports of isolation of *S. anginosus* not only from dental abscesses, but also from infective endocarditis, abscesses of brain, liver, spleen, corpus cavernosum, probably as a result of hematogenous spread (Fisher and Russell, 1993; Dugdale et al., 2013; Junckerstorff et al., 2014; Esplin et al., 2017; Finn et al., 2018; Wu and Zheng, 2020). A correlation between *S. anginosus* infections in oncological patients with esophageal cancer have been suggested (Morita et al., 2003; Rawla et al., 2017). *S*. *anginosus* was frequently isolated from the sputum of cystic fibrosis (*CF*) patients together with *Pseudomonas aeruginosa* and *Staphylococcus aureus*, the principal *CF* pathogens, and presumably was responsible for exacerbation of pneumonia (Waite et al., 2012; Whiley et al., 2014). *In vitro S. anginosus* was shown to enhance *P. aeruginosa* pathogenicity (Whiley et al., 2014; Waite et al., 2017). *S. anginosus* was also recovered from patients in co-infections with other species, e.g., *Mycobacterium tuberculosis*, and *Eikenella corrodens* (Rabuñal et al., 2009; Patel et al., 2020). All these data strongly suggest that *S. anginosus* should be viewed as an emerging opportunistic pathogen of substantial clinical importance (Reißmann et al., 2010; Pilarczyk-Zurek et al., 2022).

Streptococci use diverse strategies to adhere to, invade, and finally colonize host cells or tissues to acquire nutrition, and also to evade the defence mechanisms by suppressing the immune response (Zachary, 2017; Sitkiewicz, 2018). Numerous streptococcal proteins responsible for the adherence to eukaryotic cells have been identified. They interact with components of the connective tissue and the extracellular matrix (ECM), such as collagen, fibrinogen, fibronectin, and laminin, and also bind to salivary proteins (Nobbs et al., 2009). The attachment to host surfaces is mediated by adhesins, e.g., lectin-like adhesins, and extracellular appendages such as pili and fibrils (Nobbs et al., 2009; Schüler et al., 2012). To avoid being eliminated, streptococci trick the host immune system by hiding their antigens under thick capsules, by surviving inside macrophages, or by inducing macrophage apoptosis with pore-forming enzymes from the hemolysin group (Dinkla et al., 2007; Timmer et al., 2009; Cumley et al., 2012). Using nuclease A or DNases (Chang et al., 2011; de Buhr et al., 2014), streptococci dismantle neutrophil extracellular traps (NETs) built of chromosomal DNA and bactericidal proteins, which are released by neutrophils upon stimulation with, i.e., interleukin (IL)-8 or hydrogen peroxide (Von Köckritz-Blickwede and Nizet, 2009). Other virulence factors, such as hyaluronidase and chondroitin sulphatase, are involved in degradation of the host ECM (Asam and Spellerberg, 2014). SRRP1 (serine-rich repeat protein 1) and enolase aid the crossing of the blood – brain barrier (Sorge et al., 2009; Sun et al., 2016). Individual streptococcal species produce different repertoires of virulence factors, not all of equal importance.

Our understanding of the repertoire of virulence factors of SAG, and especially of their regulation, is still rather limited, although some mechanisms and their role in different stages of SAG infections seem firmly establish. Because of the progress in this field in recent years, the two brilliant reviews by Olson (Olson et al., 2013) on streptococcal virulence factors identified in SAG genomes *in silico*, and by Asam and Spellerberg (2014) on molecular pathogenicity of SAG species, published, respectively, in 2013 and 2014, are no longer up to date. Therefore, in the present review we will update the information on *S. anginosus*, its virulence factors and their prevalence in strains isolated from humans.

## Streptococcal virulence factors

The term “virulence factor” itself refers to a component or structure of a microorganism that helps in the establishing of an infection or in eliciting a disease. During an infection, the virulence factors of bacteria battle the protection mechanisms of the host. A streptococcal infection proceeds in three main stages: adhesion to, invasion, and colonization of the host tissues (Nobbs et al., 2009). In the genus *Streptococcus*, 189 genes coding for known and putative virulence factors have been identified (Olson et al., 2013). An analysis of 18 clinical SAG strains has revealed the presence of 55 of such factors in the SAG pan-genome, however, an individual *S. anginosus* carried only 30–34 virulence genes, of which 16 genes were found in all the SAGs analysed (Olson et al., 2013). In this review, factors with an experimentally-determined role in different aspects of *S*. *anginosus* virulence will be presented in detail, while the putative ones will only be mentioned briefly.

### Adhesion

The most important step of infection is adhesion of a pathogenic bacteria to the host tissue. This step determinates whether the bacterium will continue invasion and colonisation or not. Several host-tissue components can be exploited as targets of adhesion: fibronectin, fibrinogen, collagen, laminin and other proteins of the ECM.

#### Fibronectin-binding proteins

Fibronectin, a protein found in the ECM of connective tissue, is an important ligand used by Gram-positive cocci to adhere to host tissues. Fibronectin binding proteins (FPBs), are also called “microbial surface components recognizing adhesive matrix molecules,” or MSCRAMMs (Schwarz-Linek et al., 2004). FBPs are anchored in the bacterial cell wall, and typically comprise an N-terminal signal peptide for secretion, an LPXTG membrane anchorage motif, and a fibronectin-binding domain (Hanski et al., 1992). However, several atypical FBPs of streptococci are also known which lack a conventional secretion signal, an anchorage motif, or a typical fibronectin-binding domain, e.g., Fbp54 of *S. pyogenes* (Courtney et al., 1996). A gene encoding an atypical FBP homologue was also identified in *S. anginosus* NCTC 10713 type strain (Kodama et al., 2018). The *fbp62* gene codes for a 62.8-kDa cell-wall localized protein (Fbp62) without a signal peptide and a membrane anchorage motif. While *S. anginosus* NCTC 10713 was able to bind to immobilized fibronectin and epithelial cells of the HEp-2 (human laryngeal carcinoma) and DOK (human dysplastic keratinocyte) lines, its knock-out mutant Δ*fbp62* was not. Moreover, the *fbp62* mutation lowered mortality and abscess formation in a mouse infection model (Kodama et al., 2018). These results imply that Fbp62 is a *bona fide* virulence factor of *S. anginosus*. Earlier studies have revealed that all clinical SAG isolates recovered from abscesses, chest infection or nephritis can bind rat fibronectin, but with efficiencies varying 10-fold, especially among the *S. anginosus* strains (Willcox, 1995).

Also glyceraldehyde-3-phosphate dehydrogenase (GAPDH) of *S. pyogenes* or *S. suis*, a glycolytic enzyme, found at the cell surface of these bacteria, binds fibronectin. By showing these two unrelated activities it can be classed as a moonlighting protein [for review (Henderson and Martin, 2011)]. In addition to fibronectin, *S. pyogenes* GAPDH binds lysozyme, myosin, and actin (Pancholi and Fischetti, 1992). The fibronectin-binding function of GAPDH in *S. anginosus* has not been tested. Nevertheless, a fraction of this enzyme was detected on the cell surface and among plasminogen-binding proteins (Kinnby et al., 2008; see below). In another survey the *gapC* gene, coding for a monomer of cell-surface located GAPDH, was detected by PCR in all 22 *S. anginosus* clinical strains tested, indicating that GAPDH could act as a virulence factor also in *S. anginosus* (Chang and Lo, 2013).

#### Fibrinogen-binding proteins

Fibrinogen (Fg) is a blood plasma glycoprotein composed of two trimers of non-identical α, β, and γ chains. It plays a crucial role in hemostasis and in the innate immune system by forming insoluble fibrin clots (Herrick et al., 1999; Rubel et al., 2001). Numerous proteins of diverse Gram-positive bacteria, including streptococci can interact with Fg by distinct mechanisms [for review (Rivera et al., 2007)]. In *S. pyogenes* the M protein, its main virulence factor, binds Fg and plays an anti-phagocytic role thus enhancing bacterial survival in the host (Courtney et al., 2006). Besides Fg, the M protein can also interact with other serum proteins, such as plasminogen and immunoglobulins, and with collagen (Courtney et al., 2002). In *S. suis* muramidase-released protein (MRP), a 136-kDa cell wall-anchored surface protein, is a major fibrinogen-binding protein. A recent study showed that binding of MRP with human fibrinogen improves the viability of *S. suis* in the human blood and also favours the development of meningitis (Pian et al., 2015). Two fibrinogen-binding proteins in *S. agalactiae*, FbsA and FbsB, were shown to bind human fibrinogen and form a semi-flexible polymer-like network effectively preventing phagocytosis (Tenenbaum et al., 2005). Ability to bind human Fg was also shown for clinical *S. anginosus* strains, although the exact mechanism of Fg binding by *S. anginosus* is not yet clear (Willcox, 1995). Moreover, a whole-genome comparative analysis of seven other clinical *S. anginosus* strains identified eight proteins out of 58 with the LPXTG motif as fibrinogen-binding ones (Olson et al., 2013).

#### Laminin-binding protein

Laminins are glycoproteins of the ECM and a major component of the basement membrane; laminin binding facilitates adhesion of streptococci. The clinical *S. anginosus* strain SLEH753 isolated from infective endocarditis was shown to adhere strongly to an exposed basement membrane of human and porcine valves (mimicking native endovascular surface) in a laminin- as well as fibronectin-dependent manner (Allen et al., 2002). An 80-kDa surface lipoprotein was isolated from this strain as a putative laminin binding protein (PLBP), albeit direct binding of the purified PLBP to laminins was not shown (Allen and Höök, 2002).

#### Putative adhesion factors

A/ Serum opacity factor (SOF), an extracellular lipoproteinase, is another major fibronectin-binding protein of streptococci expressed by *ca*. 50% of invasive *S. pyogenes* strains (Courtney and Pownall, 2010). Its main function is disrupting the organization of high-density lipoproteins, which results in the formation of large water-insoluble lipid vesicles making the serum opaque. Apart from fibronectin, SOF binds other host molecules involved in bacterial adherence, such as fibrinogen and fibulin-1 (Courtney and Pownall, 2010). In *S. pyogenes* SOF is encoded by the *sof* gene. Its homologue was detected in several clinical isolates of various streptoccoci, including two of five analysed strains of SAG of group F (Reitmeyer et al., 1989).

B/ Pneumococcal surface adhesin A (PsaA) is a 37-kDa protein encoded by the *psaA* gene reported in a *S. pneumoniae* strain serotype 6B (Jado et al., 2001). This protein is likely to be involved in pneumococcal virulence, as immunization of mice with PsaA reduced the nasal load of pneumococci (Briles et al., 2000). The *psaA* homologue identical in 95% or 94% to the pneumococcal one was found in *S. mitis* and *S. oralis*, respectively (Jado et al., 2001). In *S. anginosus* type strain NCTC 10713 the *psaA* homologue is 90% identical with pneumococcal *psaA*. However, its role in SAG virulence has not been studied.

C/ Antigen I/II – the major adhesins of oral cavity streptococci are Antigens I/II (Ag I/II), cell surface-anchored proteins of *ca*. 180–210 kDa (Moisset et al., 1994). Proteins of the Ag I/II family are multifunctional, with functions varying among bacterial species and the niches occupied in the host [for review (Manzer et al., 2020)]. The first Ag I/II was characterized in *S. mutans* where it plays a crucial role in teeth colonisation. It is encoded by the *spaP* gene (also called *pac*; Russell et al., 1980). Another *S. mutans* Ag I/II-related protein, SR (salivary receptor), interacts with salivary glycoproteins adsorbed on the tooth surface (Hajishengallis et al., 1992). When present in the bloodstream, the Ag I/II proteins can modulate the immune response and induce a proinflammatory reactions (for review (Manzer et al., 2020)). Sequences coding for homologues of the Ag I/II and SR antigen were detected in two α-hemolytic *S. anginosus* strains NMH10 and PC4890 by Southern hybridization with the *spaP* gene fragments (Ma et al., 1991). Further studies are needed to confirm the presence of the Ag I/II-like antigens on the cell surface of SAGs.

D/ Pneumococcal adherence and virulence factor B (PavB), encoded by the *pavB* gene, is a surface-exposed adhesin of *S. pneumoniae*. A homologue of *pavB* has been detected in some *S. anginosus* strains. It codes for a protein 62% identical to pneumococal PavB on 56% of its length (Olson et al., 2013).

F/ Internalin A is a major invasion protein of *Listeria monocytogenes*, an intercellular pathogen, involved in the attachment to hepatocyte, epithelial, and endothelial cells and in the invasion of their vacuoles; it is encoded by *inlA* (Bierne et al., 2018). An internalin A homologue, the Slr (Streptococcus leucine-rich) protein, is produced by *S. pyogenes* (Reid et al., 2003). A gene homologous to *inlA* was also found in two of seven genomes of *S. anginosus* strains analysed. It codes for a protein 47% identical to internalin on 89% of its length (Sawyer et al., 1996; Olson et al., 2013).

G/ Fimbriae (Pili). Bacterial adhesion to surfaces and the resulting virulence is also dependent on hair-like structures located on the bacterial cell surface. In contrast to those of Gram-negative bacteria, in Gram-positive bacteria they are not well described [for review (Telford et al., 2006)]. Two types of surface appendages have been detected in Gram-positive bacteria: fimbriae (also called pili) – up to 3 μm long, flexible, thick rods (3–10 nm in diameter), and fibrils – short, thin rods (1–2 nm in diameter) extending for 70–500 nm from the bacterial surface. Historically, pili and fimbriae were discovered independently and named by two research groups. Although nowadays their names are considered synonymous (Telford et al., 2006), fimbriae seem to be related mainly to adhesion, while pili also to DNA uptake (Ottow, 1975). The pili of Gram-positive bacteria are composed of three covalently bound protein subunits carrying LPXTG sorting motif and also covalently bound to the peptidoglycan. The amino acid sequences of the pili of invasive streptococcal strains are similar to the MSCRAMM proteins involved in interaction with components of the ECM. Genes encoding pili subunits and a sortase, as well as their regulators are located in the pilus pathogenicity island organised similarly in all main groups of streptococci such as GAS, GBS, and pneumococci (Telford et al., 2006). Fibrils produced by streptococci help to bind fibronectin and better adhere to the host tissue (Telford et al., 2006). They have been identified in, e.g., *S. agalactiae*, *S. pneumoniae*, *S. pyogenes*, *S. mutans*, and in two SAG species: *S. intermedius*, and *S. constellatus* [see (Nobbs et al., 2009)].

In *S. intermedius* fimbriae are involved in saliva-mediated aggregation and associated with adherence. They are encoded by the *saf* operon, where *saf3* encodes the main pilus subunit (Yamaguchi et al., 2009). An analysis of 16 clinical strains of SAG, including six *S. anginosus* strains, identified the *saf3* gene in only four *S. intermedius* strains (Yamaguchi et al., 2009). In an earlier study the saliva-mediated aggregation was observed for 17 *S. intermedius* and two *S. constellatus* strains and for none of the six *S. anginosus* strains (Yamaguchi and Matsunoshita, 2004). It is therefore likely that *S. anginosus* does not produce fimbriae.

### Invasion and colonisation

Factors enabling bacteria to evade the host immune system and to improve the niche for living are considered important for the colonisation and invasion of host tissues.

#### Capsule

One of the first streptococcal virulence factors identified was the capsule. It is built from capsular polysaccharide (CPS) and has been detected in numerous streptococcal strains of various species: *S. pyogenes*, *S. pneumoniae*, *S. mitis*, *S. gordonii*, *S. agalactiae*, *S. suis*, and SAGs (Smith et al., 1999; Rukke et al., 2012; Toniolo et al., 2015; Skov Sørensen et al., 2016). The biochemical composition of the capsule differs between species. For example, in *S. pyogenes* capsular polysaccharide is hyaluronic acid [for review (Wessels, 2019)], and the simplest CPS of *S. pneumoniae* is a linear polymer with a repeat unit comprising two or more monosaccharides [for review (Paton and Trappetti, 2019)]. A typical *cps* cluster encoding enzymes of the CPS synthesis pathway in *S. pneumoniae* is located in the chromosome between the *dexB* and *aliA* genes, and comprises 12 to 20 genes. The capsule not only helps *S. pneumoniae* to defend its own cells against the host immune system, phagocytosis and binding by NETs, but it also contributes to pathogenesis in many ways and is absolutely required in systemic infections (Wartha et al., 2007; Hyams et al., 2010; Paton and Trappetti, 2019).

A *cps* locus similar to that of *S. pneumoniae* is often harboured by oral streptococci belonging to the groups, salivarius (*S. salivarius*), mitis (*S. mitis* and *S. oralis*), sanguinis (*S. sanguinis* and *S. gordonii*), and anginosus (*S. anginosus* and *S. intermedius*; Skov Sørensen et al., 2016). The main role of the capsule of oral streptococci is adherence to salivary components, biofilm formation, and interaction with other bacterial species; in the latter case CPS acts as a receptor for lectin-like adhesins (Cisar et al., 1995).

**Figure 2:**
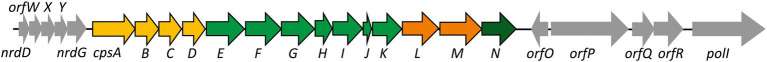
Organization of the *cps* locus of *S. anginosus*. Arrows depict genes coding for: transferases (green), regulatory proteins (yellow), polymerase and flippase (orange), mutase (dark green), other genes are in grey. Details in the text.

The structure of the locus associated with CPS synthesis has been studied in detail in type strain *S. anginosus* ATCC 33397 (NCTC 10713; Tsunashima et al., 2012; Figure 2). The region consists of 24 ORFs with 14 central genes, *cpsA* – *cpsN*, directly associated with CPS synthesis. The first four, *cpsA* – *cpsD*, are predicted to code for, respectively, a membrane-bound transcription factor for the *cps* operon, phosphotyrosine-protein phosphatase, a regulatory protein, and a tyrosine protein kinase. Based on the homology of the subsequent *cps* genes of *S. anginosus* to *cps* genes of other streptococci, they are inferred to encode transferases for undecaprenyl-phosphate glucose-1-phosphate (*cpsE*), rhamnose (*cpsF*), *N*-acetylgalactosamine (*cpsG*), *O*-acetylserine (*cpsH*), acetylgalactosamine (*cpsI*), rhamnose (*cpsJ*), and galactofuranose (*cpsK*). The following genes code for: the polysaccharide polymerase Wzy (*cpsL*) which joins the repeating units to form polysaccharide, the repeating unit transporter (flippase) Wzx (*cpsM*) engaged in transporting of the repeating units to the outer surface of the membrane and UDP-galactopyranose mutase (*cpsN*). The importance of *cpsE* for CPS production in *S. anginosus* has been verified experimentally (Tsunashima et al., 2012).

The genes upstream of *cpsA* code for: *nrdD* – ribonucleoside-triphosphate reductase, *orfW*, *orfX* and *orfY* – acetyltransferases, *nrdG* – anaerobic ribonucleoside triphosphate reductase activator protein. Downstream of *cpsN* lie genes encoding: *orfO* – a phosphoglycerate mutase family protein, *orfP* – a cell wall surface anchor family protein, *orfQ* – a DNA-binding response regulator, *orfR* – a sensor histidine kinase, and *polI* – DNA polymerase I (Tsunashima et al., 2012). The *cps* loci of *S*. *gordonii* and *S*. *sanguinis* have an organization and chromosomal localization similar to that of *S. anginosus* (Tsunashima et al., 2012).

#### Plasminogen-binding proteins: α-enolase and glyceraldehyde-3-phosphate dehydrogenase

α-Enolase is a glycolytic enzyme, but it can also be found on the surface of bacterial cells where it acts as a receptor for human plasminogen to aid invasion (Kornblatt et al., 2011). Similarly to GAPDH, α-enolase is therefore a moonlighting protein [for review (Henderson and Martin, 2014)]. α-Enolase in its receptor function has been found in *S. pneumoniae*, *S. pyogenes*, *S. suis*, *S. mutans*, and *S. iniae* (Pancholi and Fischetti, 1998; Bergmann et al., 2001; Ge et al., 2004; Esgleas et al., 2008; Membrebe et al., 2016). In *S. suis* α-enolase can also bind fibronectin and fibrinogen (Esgleas et al., 2008; Pian et al., 2015), thereby improving an antiphagocytic effect.

The streptococcal α-enolases are homo-octamers of 45-kDa monomers, each with two potential plasminogen binding sites (Cork et al., 2009; Kornblatt et al., 2011). In *S. pneumoniae* α-enolase the plasminogen binding depends on two C-terminal lysine residues and an internal plasminogen binding motif (IPM; Itzek et al., 2010). An analysis of 56 clinical strains of oral streptococci, including eight SAG strains, has shown that IPM is conserved in their α-enolases (Itzek et al., 2010). Plasminogen binding was analysed in detail in three *S. anginosus* strains of human oral origin. In one *S. anginosus* strain the plasminogen-binding proteins were identified as α-enolase and phosphoglycerate mutase; the second produced 11 plasminogen-binding proteins, of which five were identified as the glycolytic enzymes α-enolase, phosphoglycerate kinase, GAPDH, phosphoglycerate mutase, and triosephosphate isomerase; and the third – 14 proteins identified as standard glycolytic enzymes or their isoforms (Kinnby et al., 2008).

#### Streptococcal hemolysins

Hemolysins are well-described streptococcal virulence factors. Different types of hemolysins have been identified and named after the species or genus in which they were detected. Some hemolysins are produced by several species, like streptolysin S (SLS), an oxygen stable cytolytic toxin of *S. pyogenes*, *S*. *iniae*, and *S*. *constellatus*, and streptolysin O, an oxygen labile one, produced by *S*. *pyogenes*, *S. canis*, and *S. dysgalactiae* subsp. *equisimilis* (SDSE). Both of them lyse erythrocytes, leukocytes and platelets, forming holes in their membranes. Streptolysin S is a small peptide (2.7 kDa) synthesised ribosomally and extensively modified post-translationally (Nizet et al., 2000). The latter feature makes it related to the post-translationally modified bacteriocins (Molloy et al., 2011). These modifications result in the formation of aromatic thiazole and (methyl-)oxazole heterocycles prior to SLS export (Lee et al., 2008). Proteins involved in SLS production are encoded by the *sag* operon. Typically, as in *S. pyogenes*, it comprises nine genes *sagA–I*, with *sagA* encoding an SLS precursor. The *sagB* product is a 36.0-kDa protein 22% identical to a dehydrogenase that modifies McbA, the precursor of the *E. coli* bacteriocin microcin B17, one of the thiazole/oxazole-modified microcins (TOMMs). SagC (40.3 kDa) is 13% identical to the McbA cyclodehydratase, and SagD (51.6 kDa) is a component of the SagBCD complex and probably regulates its enzymatic activity (Nizet et al., 2000; Lee et al., 2008). *sagE* encodes a 25.4-kDa immunity protein localized in the membrane, similarly to the 26.2-kDa product of *sagF*. The *sagGHI*-encoded proteins are putative ABC transporters, important in extracellular transport (Nizet et al., 2000).

Streptolysin O (63,65 kDa) is a cholesterol-dependent cytolysin composed of 571 amino acids, with a 33-residues N-terminal secretion signal peptide (Kehoe et al., 1987). Other hemolysins seem to be unique to single bacterial species only, e.g., intermedilysin to *S*. *intermedius* (Nagamune et al., 1996), suilysin to *S*. *suis* (Xu et al., 2010), and pneumolysin to *S*. *pneumoniae* (Shumway and Klebanoff, 1971). Although most hemolysins show the expected hemolytic activity, there are exceptions – some pneumolysins have no hemolytic activity (Nadeem Khan et al., 2014). Pneumolysins were originally believed to be released only upon *S*. *pneumoniae* death, but later data showed that their active forms are in fact bound to the bacterial cell surface (Price and Camilli, 2009).

Notably, recent data have revealed that various streptococcal hemolysins have more sophisticated functions than simple lysis of erythrocytes. Streptolysin O suppresses the neutrophil oxidative burst, a rapid production of reactive oxygen species in response to pathogen invasion, and also helps to avoid the bacteria killing by neutrophil by blocking their degranulation, interleukin-8, and elastase secretion, which in turn suppresses the formation of NETs (Timmer et al., 2009; Uchiyama et al., 2015). Some hemolysins also affect the invasion of eukaryotic cells, as for example the cell-bound fraction of intermedilysin during invasion of human liver cell line HepG2 by *S*. *intermedius* (Sukeno et al., 2005). Also pneumolysins are involved in the *S. pneumoniae* pathogenicity regardless of their hemolytic activity, as they are engaged in biofilm formation (Kirkham et al., 2006; Shak et al., 2013).

#### Hemolysins of *Streptococcus anginosus*

As mentioned earlier, few of the *S. anginosus* strains isolated from infections are β-hemolytic. The β-hemolysin of *S. anginosus* has been identified as SLS (Asam et al., 2013; Tabata et al., 2013), no streptolysin O producing strains have been reported. SLS is synthesised as a precursor comprising a 23-amino acid signal peptide and a 30-amino acid structural peptide which is further modified post-translationally, similarly to other members of the SLS-like group of peptides (Bernheimer, 1967; Molloy et al., 2011). SLS is also classified as a member of the TOMM family. Similarly to gene clusters coding for other TOMM bacteriocins, the *sagA* gene encoding the SLS is followed by *sagB*, *sagC* and *sagD*, products of which introduce aromatic thiazole and (methyl-)oxazole heterocycles onto the SLS (Lee et al., 2008; Molloy et al., 2011). In β-hemolytic *S. anginosus* strains, the *sag* operon is composed of 10 genes *sagA-I*, including two copies of *sagA*, *sagA1* and *sagA2*, encoding SLS (Tabata et al., 2013). The genes responsible for the β-hemolytic phenotype were first identified in *S. anginosus* type strain NCTC 10713 (Tabata et al., 2013). A PCR screen of 125 clinical *S. anginosus* strains for the *sagA1* and *sagA2* genes showed that all β-hemolytic strains (18.4%; n = 23) carried the both genes, as did the type strain NCTC 10713, and all nonhemolytic strains (81.6%; n = 102) carried neither (Tabata et al., 2013). A mutant of NCTC 10713 depleted of both *sagA* genes was nonhemolytic, and introduction of either *sagA1* or *sagA2* restored the β-hemolytic activity. Also both single NCTC 10713 mutants, Δ*sagA1* and Δ*sagA2*, were still β-hemolytic (Tabata et al., 2013).

SLS of *S. anginosus* is a broad-range β-hemolysin causing lysis of red blood cells of humans, horse, sheep, cattle, rabbit and even chicken (Asam et al., 2015). SLS shows no cytotoxic activity towards THP-1 (acute monocytic leukemia) cells or human granulocytes after 2 h of treatment (Asam et al., 2015). However, after 24 h morphological changes, such as a flattened morphology and bleb formation are observed in the THP-1 and HSC-2 (human oral squamous cell carcinoma line) cell lines, presumably due to the leakage of the cellular contents following disruption of the cell membrane (Tabata et al., 2019). Those changes were shown to be SLS-depended: the β-hemolytic strain NCTC 10713 markedly decreased the HSC-2 cells survival, while NCTC 10713 depleted of both *sagA*s was not cytotoxic, demonstrating that SLS of the β-hemolytic *S. anginosus* NCTC 10713 is responsible for cytotoxicity (Tabata et al., 2019).

#### Prevalence of β-hemolytic *Streptococcus anginosus* strains

The prevalence of β-hemolytic strains of *S*. *anginosus* among natural isolates is not entirely clear. While the survey of 125 clinical strains mentioned earlier (Tabata et al., 2013) found as much as 18.4% (n = 23) of those to be β-hemolytic, this proportion was markedly lower in two other studies. An analysis of 164 strains of *S*. *anginosus* isolated from throats of 1,480 US students with pharyngitis reported only 1.2% β-hemolytic ones (*n* = 2; Fox et al., 1993), and a survey of 29 *S*. *anginosus* strains isolated from patients with skin or soft tissue infections found only 7% (*n* = 2; Summanen et al., 2009). Notably, in the latter analysis all the other strains were α-hemolytic (Summanen et al., 2009). It is worth mentioning that the analysis described by Fox et al. (1993) was performed before the molecular biology-based methods of SAG identification were elaborated. The prevalence of β-hemolytic strains among isolates from apparently healthy subjects is even smaller: among SAG strains isolated from throat swabs collected from 3,416 healthy children in India only *ca*. 0.18% (*n* = 6) isolates were β-hemolytic (Nayak et al., 2019). Similarly no β-hemolytic strains were found among 25 samples collected from non-symptomatic students in the US study (Fox et al., 1993). β-hemolysis of *S*. *anginosus* is overall rare, albeit more frequent among clinical strains isolated from infections. Still, since a vast majority of the clinical strains are non-β-hemolytic one may conclude that, while being a cytotoxin, SLS, is not a critical virulence factor of *S. anginosus*.

#### DNases

Historically, four different types of extracellular DNases, A, B, C, and D, produced by GAS have been distinguished serologically, and each strain produces at least one of them (Wannamaker, 1958; Wannamaker et al., 1967; Wannamaker and Yasmineh, 1967). DNase B, known as mitogenic factor (MF) or streptococcal pyrogenic exotoxin F (SPE-F), is chromosomally encoded by the *spd* (also known as *mf*, *speF* or *spdB*) gene and is specific for GAS (Sriskandan et al., 2000; Das et al., 2017). DNase C and DNase D are encoded on prophages by genes *spd3* and *sdaD2*, respectively (Sriskandan et al., 2000; Sumby et al., 2005). Currently, DNase A remains known only serologically and as a protein. Up till now, still other GAS DNases have been identified [for review (Remmington and Turner, 2018)], including cell-wall-anchored nuclease A encoded by *spnA* (Hasegawa et al., 2010; Chang et al., 2011). It took over 60 years from discovery of the GAS-produced extracellular DNases to the conclusion about their importance in the pathogen invasion (Sumby et al., 2005). The DNases protect *S. pyogenes* from extracellular killing by degrading DNA scaffolding of the NETs (Beiter et al., 2006; Buchanan et al., 2006; de Buhr et al., 2014). In turn, by degrading streptococcal DNA, DNases enable evading the host innate immune system by avoidance of Toll-like receptor 9 (TLR9)-mediated recognition of unmethylated CpG-rich motifs in bacterial DNA, followed by cytokine overproduction (Uchiyama et al., 2012). Extracellular DNases have been identified in *S*. *suis*, *S*. *pyogenes*, *S*. *pneumoniae*, and *S*. *agalactiae* (Hasegawa et al., 2002a; Beiter et al., 2006; Korczynska et al., 2012; Derré-Bobillot et al., 2013; de Buhr et al., 2015). Moreover, GAS, similarly to SDSE, secretes streptodornase, encoded by the *sda* gene (*sdc* in SDSE; Wolinowska et al., 1991; Hasegawa et al., 2002b).

The DNase activity was tested for 518 SAG strains from clinical specimens (Jacobs and Stobberingh, 1995). Among 307 *S*. *anginosus* isolates the majority (63%) were positive for DNase activity, and the corresponding figures for *S*. *constellatus* and *S*. *intermedius* strains were 58 and 78%, respectively. It is worth noting here that species identification was based on antigenic and biochemical tests. Different results were obtained in a survey of 128 SAG strains from sputum of *CF* patients, where among 45 isolates recognised as *S*. *anginosus* all but one (98%) were DNase producers (Grinwis et al., 2010).

#### Hyaluronidase

Hyaluronic acid is the major component of the ECM of the host connective tissue. Hyaluronan is a mediator of inflammation, however its role depends of its size: small fragments are regarded pro-inflammatory while large ones serve to suppress the inflammatory response [for review (Petrey and de la Motte, 2014)]. Hyaluronidase (hyaluronate lyase) is secreted by many streptococcal strains, including SAGs; it is an important factor facilitating the spreading of bacteria through the host tissues (Günther et al., 1996). Accordingly, secretion of hyaluronidase by *S*. *agalactiae* (GBS) has been shown to suppress the immune response and increase the intracellular survival of the bacteria in macrophages (Wang et al., 2014; Kolar et al., 2015). In GAS, GBS, and clinical strains of *S. pneumoniae* hyaluronidase also enables the use of hyaluronic acid produced by the host, or even that derived from capsules of other streptococci, for example GAS, as an alternative carbon source for growth (Rivera Starr and Cary, 2006; Marion et al., 2012). Moreover, hyaluronidase is involved in the spreading of *S*. *intermedius* by facilitating their detachment from a biofilm (Pecharki et al., 2008). The extracellular hyaluronidase produced by *S*. *pyogenes* is a protein of 99.6 kDa encoded by the *hylA* gene (Hynes et al., 2000, 2009).

Some light on the prevalence of hyaluronidase in diverse serological groups of streptococci has been shed by a large research on 614 GAS, 247 GBS, 225 group C streptococci (GCS) and 143 group G (GGS) strains (Günther et al., 1996). Among the GAS only 12.5% produced hyaluronidase, whereas in the other groups hyaluronidase positive strains were more frequent: GBS – 72.1%, GCS – 84%, and GGS – 85.5%. A survey of SAG strains (no species identified) isolated from clinical cases (n = 165) and from healthy people (n = 97) showed that on average 41% of the strains were hyaluronidase-positive, but this rate was twice as high among the clinical strains (*ca*. 50%) compared with those from a normal flora (*ca*. 25%; Unsworth, 1989). The frequency of hyaluronidase production also differs markedly for the three SAG species. In a study of 518 SAG strains recovered from clinical swabs a hyaluronidase activity was detected in only 8% (n = 25) of all *S. anginosus* strains (n = 307), both related to infections (11 of 163, 7%) and of unknown clinical importance (14 of 144, 10%; Jacobs and Stobberingh, 1995). In stark contrast, hyaluronidase producers represented 96 and 83% of *S. constellatus* and *S. intermedius* strains, respectively. Consistently, in a study of the SAG strains isolated from patients with acute dento-alveolar abscesses, none of 15 *S*. *anginosus* strains identified produced hyaluronidase (Fisher and Russell, 1993). A similar outcome was also obtained in an analysis of 157 SAG strains (64 dental plaque isolates and 91 from various other clinical sources), among which an average of 67% produced hyaluronidase (Whiley et al., 1990). However, of the 47 *S*. *anginosus* strains only 4% (n = 2) did, while 98% of *S*. *intermedius* and 88% of *S*. *constellatus* strains were hyaluronidase-positive (Whiley et al., 1990). Thus, hyaluronidase is produced by strikingly few *S*. *anginosus* strains and, therefore, appears not to be as important in their virulence as in other streptococci. However, it should be noted that in surveys mentioned above (Whiley et al., 1990; Fisher and Russell, 1993; Jacobs and Stobberingh, 1995) the SAG species were identified with antigenic and biochemical tests, prior the molecular biology-based methods have been introduced.

#### Hydrogen sulfide production

Hydrogen sulfide (H_2_S) is toxic for mammals at high concentrations. Its major effect is covalent modification of hemoglobin and its release from erythrocytes (Beauchamp et al., 1984). H_2_S is produced from L-cysteine in an α, β-elimination reaction catalysed by L-cysteine desulfhydrase (βC-S lyase, Lcd; Guarneros and Ortega, 1970; Kurzban et al., 1999). An involvement of H_2_S in progression of periodontal diseases has been proposed, especially for oral Gram-negative bacteria, the major H_2_S producers (Ratcliff and Johnson, 1999). Notably, the crude extracts from *S. anginosus* have a much higher capacity to produce hydrogen sulfide than those from other oral streptococci (Yoshida et al., 2003). The *lcd* gene encoding L-cysteine desulfhydrase from the laboratory *S. anginosus* strain FW73 was cloned in *E. coli* and the enzyme purified (Yoshida et al., 2002); exposition of sheep erythrocytes to L-cysteine and βC-S lyase resulted in the release of hemoglobin and its modification. Moreover, in BALB/c mice dental abscesses were formed after dorsal subcutaneous injection of the FW73 strain (Takahashi et al., 2011). When the mice were injected *S. anginosus* together with L-cysteine, the mean diameter of the abscesses was larger and also the level of *lcd* expression in the pus was over 15-fold higher than in mice injected with *S. anginosus* without L-cysteine. This indicated that H_2_S produced by L-cysteine desulfhydrase was responsible for the formation of the odontogenic abscesses in mouse (Takahashi et al., 2011).

#### Superantigens

Bacterial superantigens are toxins acting as T lymphocytes mitogens. They bypass antigen-presenting cells and directly stimulate the massive polyclonal proliferation of T cells, what results in the systemic release of pro-inflammatory cytokines (“cytokine storm”), including interferon-gamma (IFN-γ), tumour necrosis factor-alpha (TNFα) interleukin-beta (IL-1β), and IL-6, which in turn lead to a high fever and a toxic shock (Fraser and Proft, 2008; Wilde et al., 2021). In *S*. *pyogenes* the superantigens comprise streptococcal pyrogenic toxins (SPEs), streptococcal superantigen (SSA), and streptococcal mitogenic exotoxin Z_n_ (SMEZ_n_; Spaulding et al., 2013; Commons et al., 2014). The SPEs are divided into serotypes named A, C and G to O. SPEG and SMEZ_n_ are encoded chromosomally, in contrast to the other superantigens which are encoded by bacteriophages.

A collection of 124 β-hemolytic streptococcal strains isolated from 1,040 patients with diagnosed acute pharyngitis was tested by PCR for the presence of 11 known *S*. *pyogenes* superantigen-encoding genes (Anand et al., 2012). With the use of antigenic and biochemical tests 67 isolates (54%) were identified as *S*. *anginosus*, 38 (31%) as SDSE, and 19 (15%) as *S*. *pyogenes*. The *speC, speG*, and *smeZ* genes were detected in 100, 97.1, and 77.1% of the strains, respectively. The largest set comprising *speC*, *speG*, *speA*, *speH*, *speI*, and *smeZ* was detected in seven strains including one *S*. *anginosus*. Moreover, every *S. anginosus* strain harboured at least one gene encoding a superantigen. However, their nucleotide sequences were not determined (Anand et al., 2012). Recently, in a study of 59 β-hemolytic *S. anginosus* strains collected from endocarditis cases, the *speC* and *speG* genes as well as *sdc* and *sdaD* coding for DNases were detected in 1–8% of strains (Babbar et al., 2017). The genes were sequenced showing 100% identity with the respective *S. pyogenes* genes.

#### SAA, a novel *Streptococcus anginosus* antigen

Nitric oxide (NO) produced in response to bacterial infection has bactericidal effects [for review (Vatansever et al., 2013)]. Components of the *S. mutans* cell wall, rhamnose glucose polymers, and lipoteichoic acid from *S. sanguis* and *S. mutans* have been shown to induce NO production in macrophages (English et al., 1996; Martin et al., 1997). A similar feature has been observed for *S. anginosus* – induction of the production of both NO and inflammatory cytokines by murine peritoneal exudate cells (PEC; Sasaki et al., 2001). The agent responsible for that was purified from a culture supernatant of *S. anginosus* NCTC 10713 type strain and named *S. anginosus* antigen (SAA). SAA was then shown to stimulate NO production by PEC and accumulation of induced NO synthetase mRNA in a dose dependent manner *in vitro*. Furthermore, SAA also induced the accumulation of mRNA of TNF-α, IL–1β, and IL–6 (Sasaki et al., 2001). SAA was recognized as a tyrosine tRNA synthetase (TyrRS) belonging to the aminoacyl-tRNA synthetase family (Shimoyama et al., 2020). TyrRS could be isolated from whole-cell lysates of eight oral streptococci type strains (*S. anginosus*, *S. mutans*, *S. intermedius, S. mitis, S. sobrinus, S. gordonii, S. sanguinus,* and *S. constellatus*) but not from *S. salivarius* or *S. oralis*. It was also detected in culture supernatants of six out of seven clinical strains of *S. anginosus* from dental plaques but not in the supernatants of nine other oral streptococci. Interestingly, TyrRS has been found extracellularly uniquely in *S. anginosus*. The secretory system exporting TyrRS of *S. anginosus* outside the cell is yet to be elucidated as the protein lacks a typical N-terminal signal peptide recognised by the Sec secretion system (Shimoyama et al., 2020).

#### Signalling mediated by LuxS orthologues

Biofilm is the predominant type of growth for most bacterial species as it gives their communities a much higher resistance to changing environmental factors or antimicrobials compared to free-floating planktonic cells (Mah and O’Toole, 2001). In a process called quorum sensing numerous opportunistic pathogens produce autoinducers in response to extracellular signals to coordinate population behaviour (Miller and Bassler, 2001). Inter-species communication, frequent in both Gram-positive and Gram-negative bacteria, is based on the universal signalling molecule called autoinducer–2 (AI–2) whose synthesis requires the activity of LuxS (Vendeville et al., 2005). LuxS-mediated signalling has been identified in diverse species, including *S. mitis, S. gordonii, S. pneumoniae*, and *S. agalactiae*, therefore an attempt has been made to detect the *luxS* gene also in the *S. anginosus* type strain NCTC 10713 (Petersen et al., 2006). A PCR-amplified internal fragment of putative *luxS* showed approx. 80% nucleotide sequence identity with the *luxS* sequences of *S. pneumoniae*, *S. gordonii*, and *S. mutans*. Also, a culture supernatant of *S. anginosus* NCTC 10713 induced bioluminescence in *Vibrio harveyi* BB170, an AI-2 sensor, 5-fold more efficiently than that of its isogenic Δ*luxS* mutant. In accordance with the expected role of the putative LuxS, the *luxS* deletion decreased the ability to form biofilm between 2- and 5-fold, depending on medium used. Interestingly, under anaerobic conditions *S. anginosus* formed biofilm less efficiently than in a 5% CO_2_ aerobic atmosphere, regardless of its *luxS* status (Petersen et al., 2006). Moreover, the *luxS* mutation increased susceptibility to erythromycin and ampicillin (Ahmed et al., 2007), suggesting that the communication among bacteria could be taken advantage of as a possible target for designing a new antibacterials (Ahmed et al., 2007).

#### Other putative factors

A/ UDP-glucose pyrophosphorylase encoded by *hasC* is part of the chromosomal *hasABC* operon, essential for the synthesis of the hyaluronic acid capsule of group A streptococci (Crater et al., 1995). A homologue of *hasC* encoding a protein 86% identical to that of *S. pyogenes* has been found in all 17 SAG genomes tested, including seven *S. anginosus* strains (Olson et al., 2013); however, its role in SAG remains to be determined.

B/ Pullulanase is an enzyme degrading pullulan, a polysaccharide consisting of maltotriose units joined through α-D-1-6 glycosidic bonds. In *S. pneumoniae* pullulanase (SpuA) is a cell wall-anchored protein of 143 kDa shown to be necessary for full virulence in a mouse-lung model of infection (Bongaerts et al., 2000; Hava and Camilli, 2002). Also in *S. pyogenes* pullulanase (PulA) is a surface enzyme (129 kDa) involved in adhesion to the nasopharyngeal epithelium (Hytönen et al., 2003); it may contribute to the *S. pyogenes* virulence by its glycoprotein-binding activity and also by its potential to supply energy by carbohydrate hydrolysis. In all seven genomes of *S. anginosus* strains isolated from pulmonary and blood infections, analysed by Olsen et al. (Olson et al., 2013), a *pulA* homologue was detected coding for a protein 81% identical to the *S. pyogenes* protein along 59% of its length. However, the putative pullulanase of *S. anginosus* has not been studied yet.

C/ Streptococcal invasion locus. Another factor involved in invasion and evasion of streptococci in the host is the *sil* locus (streptococcus invasion locus). In *S. pneumoniae* it comprises five genes, *silA-silE*, which work as a quorum-sensing competence regulon. In GAS the *sil* locus has been shown to markedly increase invasiveness and fatal infections in an animal model (Hidalgo-Grass et al., 2002). Homologues of the *silA* to *silE* genes have also been identified in *S. anginosus*. The encoded proteins are identical in 52, 41, 41, 54, and 80% to those of *S. pyogenes*, respectively, and cover 98, 84, 55, 99, and 99.7% of their length (Olson et al., 2013).

D/ Two-component global regulatory system – the CsrR/CsrS regulon. In GAS the two-component system CsrR/CsrS represses several genes, e.g., those enabling the synthesis of the hyaluronic acid capsule, SLS and other toxins (Engleberg et al., 2001). The importance of CsrR/CsrS in the pathogenesis of GBS has also been reported, showing that inactivation of *csrR* increased the expression of a cluster of *cyl* genes and enhanced the hemolytic activity (Spellerberg et al., 2000; Lamy et al., 2004; Jiang et al., 2005). In SAG a *csrR* homologue encoding a protein 46% identical to CsrR of *S. pyogenes* along 99% of its length has been identified (Olson et al., 2013).

Additionally, several loci coding for other putative virulence factors have been detected in individual *S. anginosus* strains, basing on their homology to the *Streptococcus* virulence gene database (Olson et al., 2013). As already stated, *S. anginosus* is the most diverse species of the SAG group and the repertoire of its virulence factors differs substantially among strains (Olson et al., 2013).

## Conclusion

Due to the increasing number of reports on the association of *S. anginosus* with a wide variety of infections ranging from oral infections, through empyema, abscesses and pulmonary infections to systemic blood infections, especially of immunocompromised and critically ill patients, it is now considered an emerging pathogen. However, in contrast to other streptococcal pathogens, the virulence factors and their regulation in *S. anginosus* are still poorly understood. One cannot exclude that its repertoire of factors facilitating infection is much broader than currently appreciated – those listed here do not exhaust the arsenal of proteins binding the host extracellular matrix and allowing the infection caused by these bacteria to spread. We hope that this review will systematize the present knowledge on the molecular basis of *S. anginosus* virulence and attract interest to this fascinating but somewhat neglected species. Further studies on the role of these factors in the infections are essential and should contribute to a better understanding of the transmission mechanisms of *S. anginosus.* This knowledge will in turn help to develop more effective means of preventing and dealing with the wide range of infections caused by this species.

## Author contributions

AK, MS, and IK-Z created conception of the study and wrote sections of the manuscript. All authors contributed to the article and approved the submitted version.

## Funding

This work was supported by the National Science Centre under Grant 2018/29/B/NZ6/00624.

## Conflict of interest

The authors declare that the research was conducted in the absence of any commercial or financial relationships that could be construed as a potential conflict of interest.

## Publisher’s note

All claims expressed in this article are solely those of the authors and do not necessarily represent those of their affiliated organizations, or those of the publisher, the editors and the reviewers. Any product that may be evaluated in this article, or claim that may be made by its manufacturer, is not guaranteed or endorsed by the publisher.
